# EIF4A3-induced circ_0022382 promotes breast cancer cell progression through the let-7a-5p/PI3K/AKT/mTOR signaling pathway and SLC7A11 axis

**DOI:** 10.3389/fonc.2024.1476731

**Published:** 2025-01-10

**Authors:** Wei Liu, Jun Zhang, Jiawen Zhang, Yu Ye, Jianqin Zhu, Qiwen Yu, Tao Li, Xiaochun Sun, Huabiao Chen

**Affiliations:** ^1^ School of Medicine, Jiangsu University, Zhenjiang, Jiangsu, China; ^2^ HEALTH BioMed Research & Development Center, Health BioMed Co., Ltd., Ningbo, Zhejiang, China; ^3^ Department of Clinical Laboratory, The Affiliated Hospital of Yangzhou University, Yangzhou, Jiangsu, China; ^4^ School of Medicine, Ningbo University, Ningbo, Zhejiang, China

**Keywords:** breast cancer, circ_0022382, let-7a-5p, PI3K/Akt/mTOR signaling pathway, SLC7A11, disulfidptosis, EIF4A3

## Abstract

**Introduction:**

Breast cancer is one of the most common cancers in women and poses a serious threat to women's health. Circular RNAs (circRNAs) have been found to be specifically expressed in cancers and regulate the growth and death of tumor cells. The role of circRNAs in breast cancer remain unknown. In this study, we explored the impacts of circRNAs on the progression of breast cancer cells.

**Methods:**

Using bioinformatics analysis, we screened out one up-regulated circRNA in breast cancer, and its function and regulatory mechanisms were confirmed by quantitative real-time PCR, cell counting kit-8 experiment, migration assay, dual luciferase reporter assay, Kyoto Encyclopedia of Genes and Genomes enrichment analysis, cell immunofluorescence, clone formation assay, scratch wound healing experiment, RNA immunoprecipitation and subcutaneous tumor-bearing experiments.

**Results:**

Circ_0022382 was highly expressed in breast cancer cell lines MDA-MB-231, MCF-7 as well as breast cancer tissues, and promoted the proliferative and migratory capacity of breast cancer cells. In terms of regulatory mechanisms, circ_0022382 activated PI3K/AKT/mTOR signaling pathway and SLC7A11 by sponging let-7a-5p, while knockdown of circ_0022382 contributed to the occurrence of disulfidptosis. In addition, EIF4A3 promoted the expression of circ_0022382 in MDA-MB-231 and MCF-7. Consistently, knockdown of circ_0022382 inhibited the growth of breast cancer cells *in vivo*.

**Discussion:**

Circ_0022382 and its related molecules may be effective targets for diagnosis or targeted therapy of breast cancer.

## 1 Introduction

Breast cancer has a high incidence and mortality among women (1). Though advances in treatment approaches have benefited many breast cancer patients, drug resistance remains due to the genomic instability inherent of the tumor itself (2, 3). Circular RNAs (circRNAs), as non-coding RNA, were first thought to be meaningless and byproducts of the gene-splicing process (4). However, with the widespread development of next-generation sequencing in clinical diagnosis and treatment, circRNAs have been found to be specifically expressed in many cancers and affect the proliferation, metastasis, drug resistance and immune response of tumor cells (5, 6). In addition, circRNAs with closed loop structure are more stable than linear RNAs. Therefore, circRNAs could act as stable and specific molecular markers of cancers. Mechanically, most of the circRNAs such as circ_104348 and circ_001783 are mainly located in the cytoplasm and exert regulatory functions by sponging microRNAs (7, 8), then regulate signaling pathways such as PI3K/AKT/mTOR signaling pathway (9). Recent studies reported correlations between circRNAs and various types of cell death such as apoptosis and ferroptosis in breast cancer (10, 11). Disulfidptosis is a type of cell death arising from accumulated cystine within cells with high expression of SLC7A11 (12, 13). However, the relationship between circRNAs and disulfidptosis in breast cancer remains unknown. In the present study, bioinformation analysis was carried out in the GEO database, which found that circ_0022382 was highly expressed in breast cancer. For mechanism of circ_0022382, we revealed that knockdown of circ_0022382 inhibited the proliferation and migration of breast cancer cells via sponging let-7a-5p, down-regulated p-AKT and SLC7A11, promoted the occurrence of disulfidptosis. RNA-binding proteins such as AUF1 and EIF4A3 were reported to induce the production of circRNAs (14, 15), in this study, we also demonstrated EIF4A3 could promote the production of circ_0022382 in breast cancer.

## 2 Materials and methods

### 2.1 Clinical samples and cells

Three paired human breast cancer tissues and adjacent tissues were obtained from the Department of Oncology Surgery (Thyroid & Breast), Affiliated Hospital of Jiangsu University. The study was approved by the Ethics Committee of Jiangsu University. Breast cancer cells including MDA-MB-231, MCF-7, normal breast epithelial cell MCF-10A and 293T were purchased from American Type Culture Collection (Manassas, VA, USA).

### 2.2 Bioinformatics analysis

Searching for the keywords “breast cancer”, “circRNA” and “miRNA” in the GEO database (https://www.ncbi.nlm.nih.gov/geo/), selected the corresponding datasets for “circRNA” and “miRNA”, the threshold of |*log_2_ (fold change, FC)*|>2 and *adj. p*<0.05 was set and perform differential analysis of GSE165884, similarly, the threshold of |*log_2_ (FC)*|>1.4 and *adj. p*<0.05 was set and perform differential analysis of GSE45498, GEO2R (https://www.ncbi.nlm.nih.gov/geo/geo2r/) was a part of GEO database to analyze and visualize the differential genes of samples, the potential miRNAs combined with circRNA were predicted by ENCORI (https://starbase.sysu.edu.cn/), and these miRNAs and down-regulated miRNAs were intersected by Venn diagram(https://bioinformatics.psb.ugent.be/webtools/Venn/), and the circRNA combined with the intersecting miRNA was taken as the research object. DAVID v6.8 (https://david-d.ncifcrf.gov/), Wei Sheng Xin platform (https://www.bioinformatics.com.cn/login/) was used to perform and visualize the results of enrichment analysis of KEGG signaling pathways on the basis of mRNAs bound by miRNA, CircInteractome (https://circinteractome.nia.nih.gov/) was used to predict the RNA-binding proteins combined with circRNA.

### 2.3 RNA extraction and quantitative real-time PCR

Total RNA was extracted from cells according to the instructions for Trizol reagent (Thermo Fisher Scientific, Carlsbad, CA, USA) and we measured the concentration using a Nanodrop 1000 ultramicro spectrophotometer (NanoDrop, Wilmington, DE, USA). For quantitative analysis of mRNA, we first reverse transcribed total RNA into cDNA according to the mRNA reverse transcription reagent instructions (Vazyme Biotech, Nanjing, China; R323-01), and then amplified the cDNA according to the qPCR instructions (Vazyme; Q711), using *GAPDH* as the internal reference, the primer sequences can be seen in the **Table 1**. For quantitative analysis of miRNA, the miRNA was first specifically reverse transcribed into cDNA using the stem loop method according to the miRNA reverse transcription reagent instructions (Vazyme; MR101-02). Then, the cDNA was amplified according to the qPCR instructions (Vazyme; MQ101-02), using U6 as the internal reference, the primer sequences can be seen in the **Table 1**. The expression of circ_0022382, let-7a-5p and SLC7A11 was measured after siRNA transfection for 36 h respectively.

**Table 1:** qRT-PCR primer sequences and transfection primer sequences.

	Forward (5’-3’)	Reverse (5’-3’)
Circ_0022382	GATTCCTACCCTCATCACGGC	GAAGGCGCGGAAGGCAT
GAPDH	AATGGGCAGCCGTTAGGAAA	GCGCCCAATACGACCAAATC
SLC7A11	TGTGTGGGGTCCTGTCACTA	CAGTAGCTGCAGGGCGTATT
Si-circ_0022382-1	CACUUAAAGGAUGCCUUCCTT	GGAAGGCAUCCUUUAAGUGTT
Si-circ_0022382-2	UUAAAGGAUGCCUUCCGCGTT	CGCGGAAGGCAUCCUUUAATT
Let-7a-5p	GCGCGTGAGGTAGTAGGTTGT	AGTGCAGGGTCCGAGGTATT
U6	CTCGCTTCGGCAGCACA	AACGCTTCACGAATTTGCGT
Si-EIF4A3	CGAGCAAUCAAGCAGAUCATT	UGAUCUGCUUGAUUGCUCGTT
Let-7a-5p mimic	UGAGGUAGUAGGUUGUAUAGUU	CUAUACAACCUACUACCUCAUU
Let-7a-5p inhibitor	AACUAUACAACCUACUACCUCA	
LV-shcirc_0022382	GCCACTTAAAGGATGCCTTCC	

### 2.4 Cell transfection

On the day before transfection, MDA-MB-231 and MCF-7 in the logarithmic growth phase were inoculated into a 6-well plate at a density of 1 × 10^5^ cell/mL. The next day, when the cell density had reached approximately 70%, cells were transfected with siRNA or miRNA mimic respectively, co-transfected with si-NC and let-7a-5p inhibitor or si-circ_0022382 and let-7a-5p inhibitor. specific procedures can be seen in the instructions for Lipofectamine^TM 2000^ (Thermo Fisher Scientific), the sequences of transfection primers can be seen in the **Table 1**.

### 2.5 Cell counting kit-8 experiment

MDA-MB-231 and MCF-7 cells transfected with si-NC or si-circ_0022382, co-transfected with si-NC and let-7a-5p inhibitor or si-circ_0022382 and let-7a-5p inhibitor for 24 h, 48 h and 72 h were digested with trypsin respectively and inoculated into 96-well plates at a cell density of 5 × 10^4^ cells/mL. After 24 h, 10 μL of Cell Counting Kit-8 reagent (Biosharp, Beijing, China) was added to each well and optical density was measured by an enzyme-linked immunosorbent assay reader at a wavelength of 450 nm.

### 2.6 Clone formation experiment

After transfecting si-circ_0022382 for 24 h, MDA-MB-231 and MCF-7 were digested with trypsin respectively and inoculated at a density of 500 cells per well into a 6-well plate and cultured in a CO_2_ incubator. The medium was changed every 3 days. When the number of cells in the clone group under the microscope exceeded 50, cell culture was stopped. The original culture medium was discarded and cells were washed three times with PBS, followed by addition of 1 mL 4% paraformaldehyde fixative for 20 min. The fixative was discarded, the cells were washed three times with PBS, and 1 mL of 0.1% crystal violet staining solution was added to each well for 20 min. The staining solution was discarded and the cells were washed three times with PBS before taking photos.

### 2.7 Scratch wound healing experiment

After transfecting si-circ_0022382 for 24 h, MDA-MB-231 and MCF-7 were digested with trypsin respectively and inoculated into 6-well plates, two days later, the cells were overgrown. A scratch was made across the cell layer using a 200 μL pipette. The culture medium was discarded and the cells were washed three times with PBS. DMEM nutrient solution with 2% FBS was then added and the cells were photographed and recorded as 0 h. After 24 h, the medium was removed and the cells were photographed again and recorded as 24 h.

### 2.8 Migration assay

MDA-MB-231 and MCF-7 cells transfected with si-NC or si-circ_0022382, co-transfected with si-NC and let-7a-5p inhibitor or si-circ_0022382 and let-7a-5p inhibitor for 24 h were digested with trypsin respectively and resuspended in serum-free high-glucose DMEM cell culture medium. The cells were inoculated into Transwell chambers at a density of 1 ×10^5^ cells/well, and the cells were placed in 600 μL of high-sugar DMEM cell culture medium containing 10% fetal bovine serum. After 12 h, the Transwell chambers were removed and washed 3 times with PBS. We used a cotton swab to wipe-off any cells that had not penetrated through the Transwell chamber, then fixed the inserts in 4% paraformaldehyde solution for 30 min. The fixative was removed and the cells were washed 3 times with PBS. Finally, crystal violet solution was used to stain the cells at room temperature for 30 min before washing 3 times with PBS, cleaning the remaining substances and wiping the upper chamber cells clean with a cotton swab. After drying at room temperature, we imaged and counted the migrating cells on the basolateral side of the chamber by Image J.

### 2.9 Dual luciferase reporter assay

Co-transfect circ_0022382 dual luciferase reporter vector (wild type plasmid or mutant type plasmid) and let-7a-5p (negative control sequence or overexpression sequence), judge whether let-7a-5p could bind to circ_0022382 or not from luciferase density, if luciferase density decline, let-7a-5p could interfere the expression of circ_0022382 plasmid, indicating that let-7a-5p could bind to circ_0022382. Among of them, mutant type plasmid was used for positive control, negative control sequence was used for negative control. Specific procedures can be seen in the instructions for the Dual Luciferase Reporter Assay Kit (Vazyme; DL 101-01).

### 2.10 RNA immunoprecipitation

EIF4A3 protein and the genes that interact with EIF4A3 could be pulled down together by magnetic beads containing the EIF4A3 antibody_(AiFang; AF301768), and the final concentration of EIF4A3 antibody stock in solution is 1 : 40, the specific procedure of RNA immunoprecipitation follows the instruction of the RIP reagent kit (Merck, DARMSTADT; 17-701).

### 2.11 Western blot

After lysing with the cell lysis buffer (LEAGENE, Beijing, China; PS0013), the cell lysate was obtained by scraping and centrifuged at 12000 g rotational speed. The supernatant was separated and 5 × loading buffer was added at a ratio of 4 : 1, and 2 μL of PMSF was added at the same time to prevent protein degradation. The total protein extract was divided into protein bands of different molecular weights by electrophoresis and membrane transfer, and then the membranes containing the different bands were blocked with 5% skim milk. The membranes were immunoblotted with primary antibodies, including anti-p-AKT_(Cell Signaling Technology; 4060T), anti-SLC7A11_(Abclonal, Wuhan; A2413), anti-EIF4A3_(AiFang; AF301768) and HRP-conjugated anti-β-actin_(Santa Cruz; sc-47778). All primary antibody stocks were diluted with TBST at a dilution ratio of 1 : 1000 and incubated with the membranes at 4°C overnight. The membranes were then washed with TBST three times and incubated for 2 h at room temperature in secondary antibody stock solution diluted in TBST at a dilution ratio of 1 : 2000. The membranes were washed in TBST three times and placed on the ECL (Vazyme; E412-02-AA) in developer (GE, Boston, MA, USA; LAS4000Mini) for imaging.

### 2.12 Lentiviral transfection and subcutaneous tumor injection

MDA-MB-231 cells were inoculated into 6-well plates, and when the cell density reached 30%, the culture medium was discarded and washed with PBS, and a lentiviral transfection mixture consisting of 2 mL of DMEM nutrient solution and 20 μL of lentiviral solution (LV-NC or LV-shcirc_0022382) was added to each well, gently mixed and placed in the incubator to continue culture for 24 h, then the culture medium was replaced after 24 h, one part of cells were used to detect the knockdown efficiency in MDA-MB-231 cells transfected with LV-shcirc_0022382, another part of cells were digested and resuspended with PBS for subcutaneous tumor-bearing experiments. Ten 4-week-old female BALB/C nude mice weighing between 15 g and 20 g were used for subcutaneous tumor-bearing experiments. 1×10^6^ cells were injected into the back of each mouse and the tumor size was measured every 3 days. Two weeks later, the mice were euthanatized by cervical dislocation, the tumors were photographed and weighed. Tumor volume = 0.52 × width^2^ (mm^2^) × length (mm). The sequence of LV-shcirc_0022382 can be seen in the **Table 1**.

### 2.13 Immunohistochemistry

Prepared the tumor tissues into paraffin slides, which were deparaffinized and hydrated with different concentrations of xylene and ethanol solutions, washed the slides with water and placed them in boiling citrate retrieval solution for antigen retrieval, soaked the slides in H_2_O_2_ after washing with PBS, washed the slides with PBS and added goat serum to the slides, incubated for 1 h at room temperature, added the primary antibody dilution to the slides, i.e, anti-Ki-67 (AiFang; AF300540), anti-SLC7A11 (Proteintech, Wuhan, China; 26864-1-AP), or anti-p-AKT (Cell Signaling Technology), incubated overnight at 4°C, washed the slides with PBS, added the secondary antibody dilution to the slides, i.e, Cy3-IgG (Abclonal, Wuhan, China; AS007), incubated for 1 h at room temperature, washed the slides with PBS and add HRP-Streptavidin, incubated for 30 min at room temperature, washed the slides with PBS and add DAB chromogenic solution, incubated for 2 min at room temperature, washed the slides with water and add hematoxylin, incubated for 1 min at room temperature, washed the slides with water, added different concentrations of ethanol and xylene solutions.

### 2.14 Cellular immunofluorescence

MDA-MB-231 and MCF-7 cells transfected with si-NC or si-circ_0022382, co-transfected with si-NC and let-7a-5p inhibitor or si-circ_0022382 and let-7a-5p inhibitor for 24 h were digested with trypsin and inoculated into crawling slides in 24-well plates for 24 h, then the culture medium was discarded and the cells were washed three times with PBS. The cells were fixed in 4% paraformaldehyde for 15 min and then washed three times in PBS. Then, the cells were permeabilized with 0.5% Triton X-100 for 20 min. The cells were washed three times in PBS and blocked with goat serum at room temperature for 30 min. After discarding the blocking solution, primary antibody, i.e., anti-p-AKT (Cell Signaling Technology), or anti-SLC7A11 (Proteintech) was added. The cells were incubated overnight at 4°C, the primary antibody was removed and the cells were washed three times with PBS, then incubated with Cy3-conjugated secondary antibody (Abclonal; AS007) at room temperature for 1 h. The cell slides were inverted onto anti-fluorescence quencher containing DAPI, and images were captured under a confocal microscope.

### 2.15 Glucose concentration detection

After transfecting si-circ_0022382 for 24 h, the culture medium supernatant of MDA-MB-231 and MCF-7 were collected respectively. The glucose concentration in the cell supernatant was detected according to the glucose kit (Nanjing Jiancheng Bioengineering Institute, Nanjing; A154-2-1).

### 2.16 Detection of the concentrations of NADPH and NADP^+^

After transfecting si-circ_0022382 for 24 h, MDA-MB-231 and MCF-7 were digested with trypsin and collected respectively. The ratio of the concentrations of NADPH and NADP^+^ were detected according to the kit (Nanjing Jiancheng Bioengineering Institute, Nanjing; A115-1-1).

### 2.17 Statistical analysis

Except for clone formation experiment, scratch wound healing experiment and cellular immunofluorescence, other experiments were performed three times, whose data were parametric and GraphPad Prism 8 software (GraphPad Software, San Diego, CA, USA) was used to analyze the data, which were expressed as mean ± Standard error of the mean (SEM). The significance of differences between the two groups was determined using the Student’s t-test, and one-way analysis of variance was used to compare multiple groups.

## 3 Results

### 3.1 Circ_0022382 is highly expressed in breast cancer and promotes proliferation and migration of breast cancer cells

**Figure 1:**
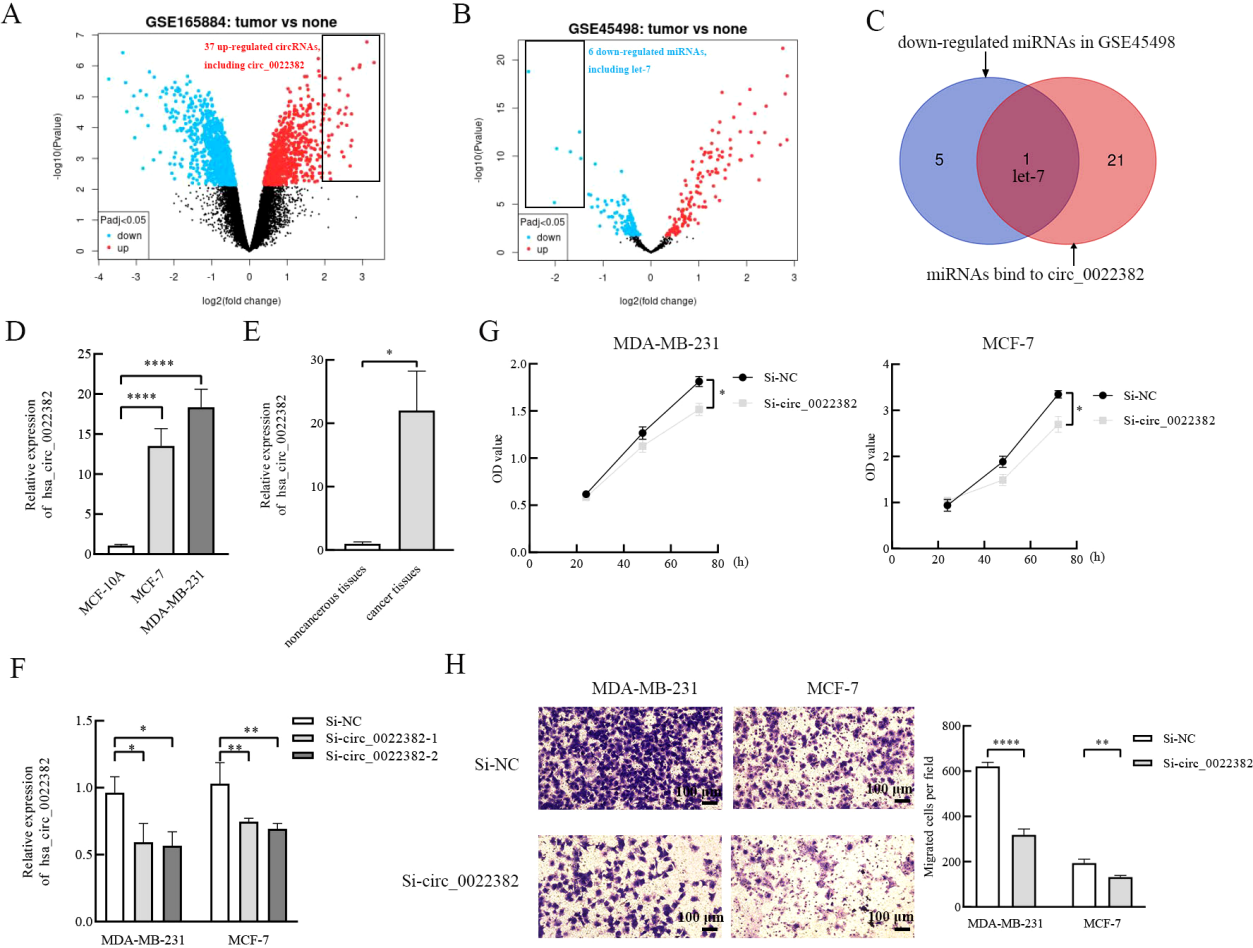
The highly expressed circ_0022382 screened from the GEO database promotes the proliferation and migration of breast cancer cells. **(A)** The volcano plot of GSE165884 was generated by GEO2R, red scatter points showed circRNAs that are up-regulated in breast cancer, including circ_0022382. **(B)** The volcano plot of GSE45498 was generated by GEO2R, blue scatter points showed miRNAs that are down-regulated in breast cancer, including let-7. **(C)** The intersection of miRNAs bind to circ_0022382 and down-regulated miRNAs was generated by Venn diagram. **(D)** QRT-PCR was used to measure the expression of circ_0022382 in MDA-MB-231, MCF-7 and MCF-10A. ****p<0.0001 vs. MCF-10A group. Data are the means ± SEM of three independent experiments. **(E)** QRT-PCR was used to measure the expression of circ_0022382 in three pairs of breast cancer and adjacent noncancerous tissues. *p<0.05 vs. adjacent noncancerous tissue group. Data are the means ± SEM of three independent experiments. **(F)** Knockdown efficiency of si-circ_0022382-1 and si-circ_0022382-2 in MDA-MB-231 and MCF-7 were measured by qRT-PCR. *p<0.05, **p<0.01 vs. Si-NC group. Data are the means ± SEM of three independent experiments. **(G)** Cell Counting Kit-8 (CCK8) was used to detect speed of proliferation in MDA-MB-231 and MCF-7 cells transfected with si-NC or si-circ_0022382-1 (si-circ_0022382) for 24 h, 48 h and 72 h. *p<0.05 vs. Si-NC group. Data are the means ± SEM of three independent experiments. **(H)** Migration assay was used to detect numbers of migrated MDA-MB-231 and MCF-7 cells transfected with si-NC or si-circ_0022382 for 24 h. **p<0.01, ****p<0.0001 vs. Si-NC group; scale bar = 100 μm. Data are the means ± SEM of three independent experiments.

Firstly, we roughly screened up-regulated circRNAs in breast cancer from GEO database, GSE165884 is a GEO dataset related to breast cancer and circRNA, by performing differential analysis of circRNAs, the volcano plot was drawn by GEO2R, which showed up-regulated and down-regulated circRNAs in breast cancer tissues respectively, and there were 37 up-regulated circRNAs at the threshold of |*log_2_ (FC)*|>2 and *adj. p*<0.05, including circ_0022382 (**Figure 1A**). Secondly, since most of circRNAs act as miRNA sponges, we further narrowed the scope of circRNAs screened in the first step, consistently, we screened down-regulated miRNAs in breast cancer from GEO database, the volcano plot was drawn by GEO2R in GSE45498 to reveal differential expressed miRNAs in breast cancer tissues, and there were 6 down-regulated miRNAs at the threshold of |*log_2_ (FC)*|>1.4 and *adj. p*<0.05, including let-7 (**Figure 1B**). By predicting miRNAs bound by circRNAs from the first step and intersecting with miRNAs from the second step (**Table 2**), we revealed an intersection between miRNAs bound by circ_0022382 and 6 down-regulated miRNAs, i.e, let-7, so we took circ_0022382 as the research object (**Figure 1C**). Then, expression of circ_0022382 was evaluated by qRT-PCR in both breast associated cells and tissues, the expression of circ_0022382 in breast cancer cells (MDA-MB-231 and MCF-7) were significantly higher than in MCF-10A (*p*<0.0001; **Figure 1D**), breast cancer tissues had higher expression of circ_0022382 than in adjacent noncancerous tissues (*p*<0.05; **Figure 1E**). In addition, we explored effects of circ_0022382 on biological functions of breast cancer cells by interfering with circ_0022382 expression, knockdown efficiency of two siRNAs (si-circ_0022382-1 and si-circ_0022382-2) was evaluated by qRT-PCR, which found two siRNAs could both effectively knock down circ_0022382 expression in MDA-MB-231 and MCF-7 (*p*<0.05 or *p*<0.01; **Figure 1F**). Then, si-circ_0022382-1 was chosen for subsequent experiments due to si-circ_0022382-1 has less aggregated G or C bases at the 3’ end (**Table 1**). CCK8 was performed to evaluate cell viability, the results showed the speed of cell proliferation was significantly slower after transfection with si-circ_0022382 (*p*<0.05; **Figure 1G**). Meanwhile, migration experiment was performed to evaluate cell migration, the results showed numbers of migrated cells were also significantly reduced after transfection with si-circ_0022382 (*p*<0.01 or *p*<0.0001; **Figure 1H**).

**Table 2:** miRNAs bind to circ_0022382 and down-regulated miRNAs in GSE45498.

down-regulated miRNAs at the threshold of |log_2_ (FC)|>1.4 and adj. p<0.05 in GSE45498	the miRNAs that bind to circ_0022382
hsa-miR-548	hsa-miR-214-3p
hsa-miR-125	hsa-miR-761
hsa-miR-145	hsa-miR-3619-5p
hsa-miR-204	hsa-miR-4640-5p
hsa-miR-199	hsa-miR-4726-5p
hsa-let-7	hsa-miR-4761-5p
	hsa-miR-3127-5p
	hsa-miR-3918
	hsa-miR-670-3p
	hsa-miR-766-5p
	hsa-miR-665
	hsa-miR-4640-5p
	hsa-miR-6720-5p
	hsa-miR-6512-3p
	hsa-miR-5586-5p
	hsa-let-7b-5p
	hsa-let-7c-5p
	hsa-miR-98-5p
	hsa-let-7i-5p
	hsa-let-7a-5p
	hsa-let-7e-5p
	hsa-let-7f-5p
	hsa-let-7g-5p
	hsa-let-7d-5p
	hsa-miR-4500
	hsa-miR-3614-5p
	hsa-miR-3187-3p
	hsa-miR-552-3p
	hsa-miR-4458
	hsa-miR-624-3p

### 3.2 Circ_0022382 promotes the proliferation and migration of breast cancer cells by sponging let-7a-5p

**Figure 2:**
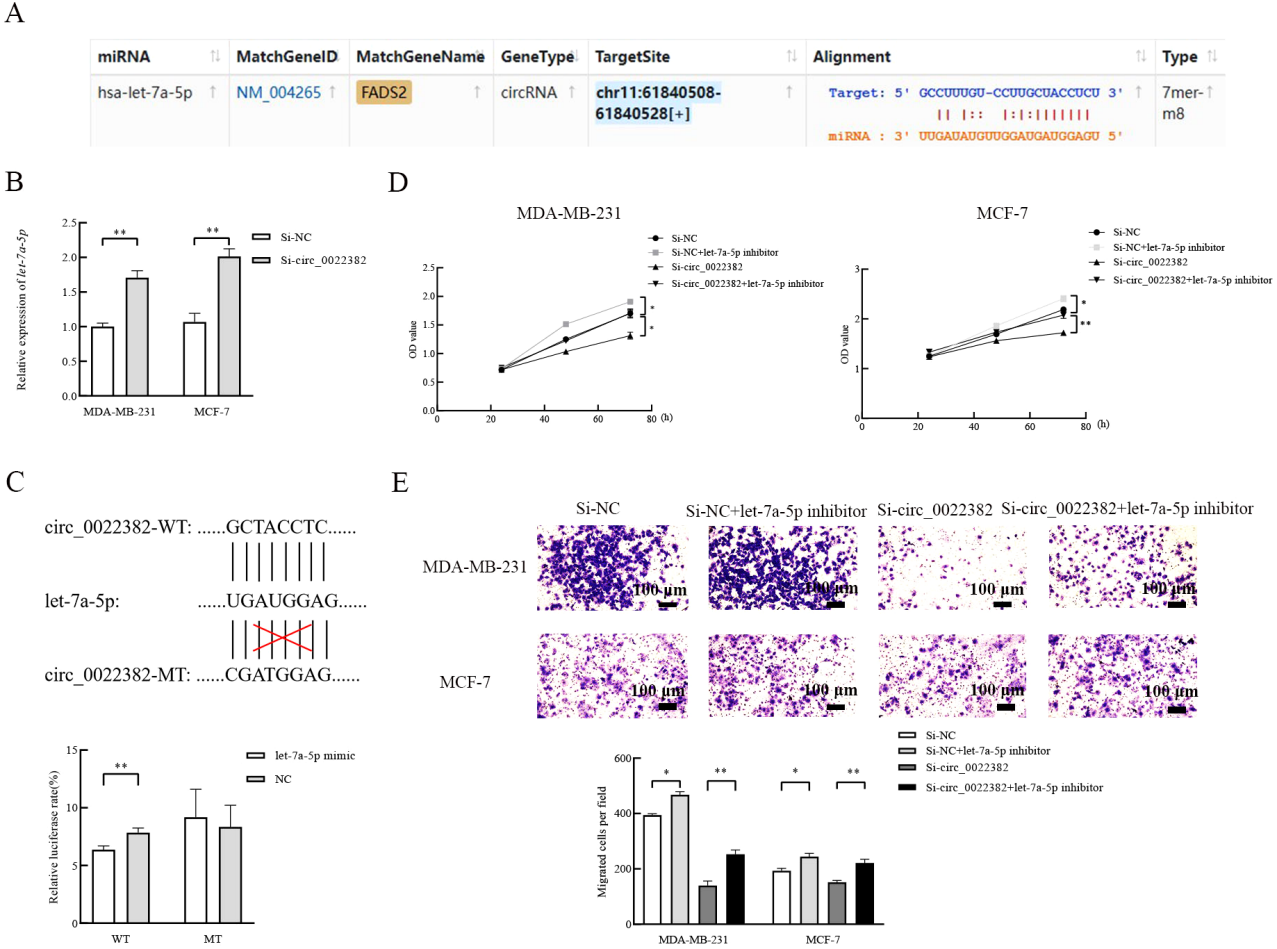
Let-7a-5p is a direct target to affect the proliferation and migration of breast cancer cells. **(A)** Let-7a-5p was predicted as a target of circ_0022382 by ENCORI. **(B)** QRT-PCR was used to measure the expression of let-7a-5p in MDA-MB-231 and MCF-7 cells transfected with si-NC or si-circ_0022382. **p<0.01 vs. Si-NC group. Data are the means ± SEM of three independent experiments. **(C)** Dual-luciferase reporter assay was used to measure the luciferase of circ_0022382 wild type plasmid (WT) and circ_0022382 mutant type plasmid (MT) in 293T cells transfected with negative control (NC) or let-7a-5p mimic. **p<0.01 vs. NC group. Data are the means ± SEM of three independent experiments. **(D)** CCK8 was used to detect speed of proliferation in MDA-MB-231 and MCF-7 cells transfected with si-NC or si-circ_0022382, co-transfected with si-NC and let-7a-5p inhibitor or si-circ_0022382 and let-7a-5p inhibitor for 24 h, 48 h and 72 h. *p<0.05, **p<0.01 vs. Si-NC group or Si-circ_0022382 group. Data are the means ± SEM of three independent experiments. **(E)** Migration assay was used to detect numbers of migrated MDA-MB-231 and MCF-7 cells transfected with si-NC or si-circ_0022382, co-transfected with si-NC and let-7a-5p inhibitor or si-circ_0022382 and let-7a-5p inhibitor for 12 h. *p<0.05, **p<0.01 vs. Si-NC group or Si-circ_0022382 group; scale bar = 100 μm. Data are the means ± SEM of three independent experiments.

In terms of mechanism, miRNA sponges have been studied the most (16), the above results showed that let-7 were potential targets of circ_0022382, so we screened the miRNA candidate from let-7 homologs by the lowercase letters of its suffix, surprisingly, ENCORI showed that let-7a-5p could bind to circ_0022382 and the binding capacity could reach 7 mer to 8 mer (**Figure 2A**). Let-7a-5p expression significantly increased after transfection with si-circ_0022382 in MDA MB-231 and MCF-7 (*p*<0.01; **Figure 2B**). Moreover, we performed dual luciferase reporter assay to further confirm this result, by co-transfecting let-7a-5p mimic and circ_0022382 wild-type plasmid with luciferase reporter, the fluorescence intensity in 293T cells significantly reduced, which indicated let-7a-5p had binding sites on circ_0022382 wild-type plasmid, on the contrary, the inhibitory effect of let-7a-5p mimic on fluorescence intensity could not be reflected when circ_0022382 plasmid was mutated (*p*<0.01; **Figure 2C**). Then, we tested whether circ_0022382 promoted the proliferation and migration of breast cancer cells by sponging let-7a-5p. The results of CCK8 showed the speed of cell proliferation was significantly faster after co-transfection with si-NC and let-7a-5p inhibitor, co-transfection with si-circ_0022382 and let-7a-5p inhibitor could rescue the effect of si-circ_0022382 on the proliferation (*p*<0.05 or *p*<0.01; **Figure 2D**), we observed similar effect of let-7a-5p inhibitor from migration experiment (*p*<0.05 or *p*<0.01; **Figure 2E**). Therefore, the above results suggested that circ_0022382 and let-7a-5p had opposite effect, circ_0022382 could interact with let-7a-5p to affect the proliferation and migration of breast cancer cells.

### 3.3 Circ_0022382 regulates PI3K/AKT/mTOR signaling pathway and SLC7A11 by sponging let-7a-5p

**Figure 3:**
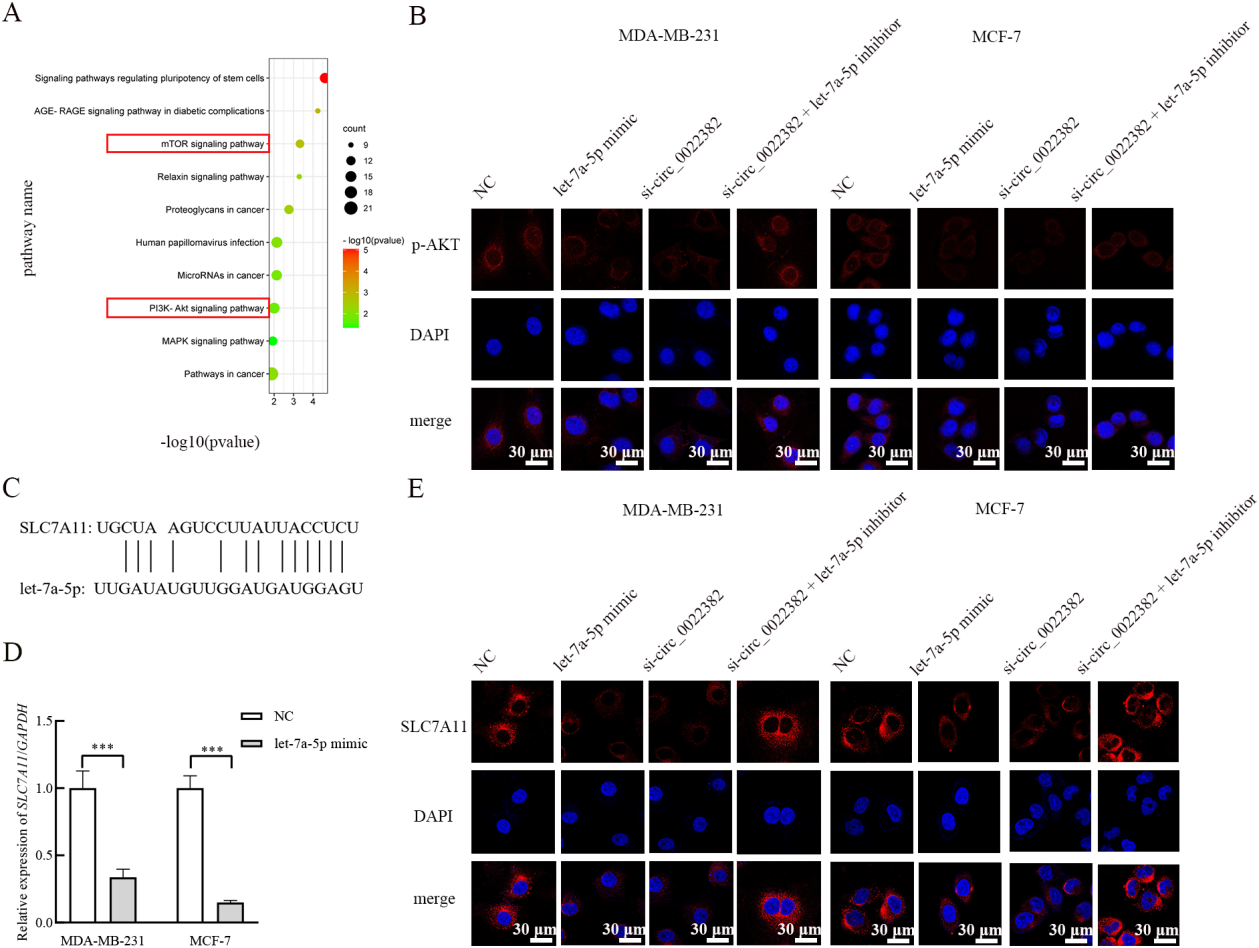
The enrichment of PI3K/AKT/mTOR signaling pathway and SLC7A11 is regulated by circ_0022382/let-7a-5p axis. **(A)** The top 10 enriched KEGG signaling pathways of downstream target genes of let-7a-5p were visualized through bubble plots. **(B)** The luciferase density of Cy3-P-AKT in MDA-MB-231 and MCF-7 cells transfected with negative control (NC), let-7a-5p mimic, si-circ_0022382 respectively, co-transfected with si-circ_0022382 and let-7a-5p inhibitor was verified by cellular immunofluorescence; scale bar = 30 μm. **(C)** SLC7A11 was predicted as a target of let-7a-5p by ENCORI. **(D)** QRT-PCR was used to measure the expression of SLC7A11 mRNA in MDA-MB-231 and MCF-7 cells transfected with NC or let-7a-5p mimic. ***p<0.001 vs. NC group. Data are the means ± SEM of three independent experiments. **(E)** The luciferase density of Cy3-SLC7A11 in MDA-MB-231 and MCF-7 cells transfected with NC, let-7a-5p mimic, si-circ_0022382 respectively, co-transfected with si-circ_0022382 and let-7a-5p inhibitor was verified by cellular immunofluorescence; scale bar = 30 μm.

As shown in the bubble plots, the downstream mRNAs of let-7a-5p were enriched in signaling pathways involved in cancer progression, i.e, mTOR signaling pathway, PI3K-AKT signaling pathway (**Figure 3A**). To further confirm this enrichment, expression of signaling pathway-related proteins was detected by cellular immunofluorescence, the results showed p-AKT expression was reduced after overexpressing let-7a-5p, while co-transfection with si-circ_0022382 and let-7a-5p inhibitor could rescue effects of si-circ_0022382 on the p-AKT expression (**Figure 3B**). Moreover, SLC7A11 was reported to be related to PI3K/AKT/mTOR signaling pathway in cancers, such as gastric cancer (17), glioma (18), pancreatic carcinoma (19), neuroendocrine tumors (20), but the interaction has not been clarified in breast cancer. To confirm the relationship, firstly we tested whether let-7a-5p regulated SLC7A11, as shown in ENCORI, let-7a-5p could bind to SLC7A11 (**Figure 3C**), the expression of SLC7A11 mRNA in MDA-MB-231 and MCF-7 transfected with let-7a-5p mimic was significantly decreased (*p*<0.001; **Figure 3D**). Moreover, the results of cellular immunofluorescence showed SLC7A11 protein expression was also reduced after overexpressing let-7a-5p, while co-transfection of si-circ_0022382 and let-7a-5p inhibitor could rescue effects of si-circ_0022382 on the SLC7A11 expression (**Figure 3E**).

### 3.4 Knockdown of circ_0022382 contributes to SLC7A11-mediated disulfidptosis

**Figure 4:**
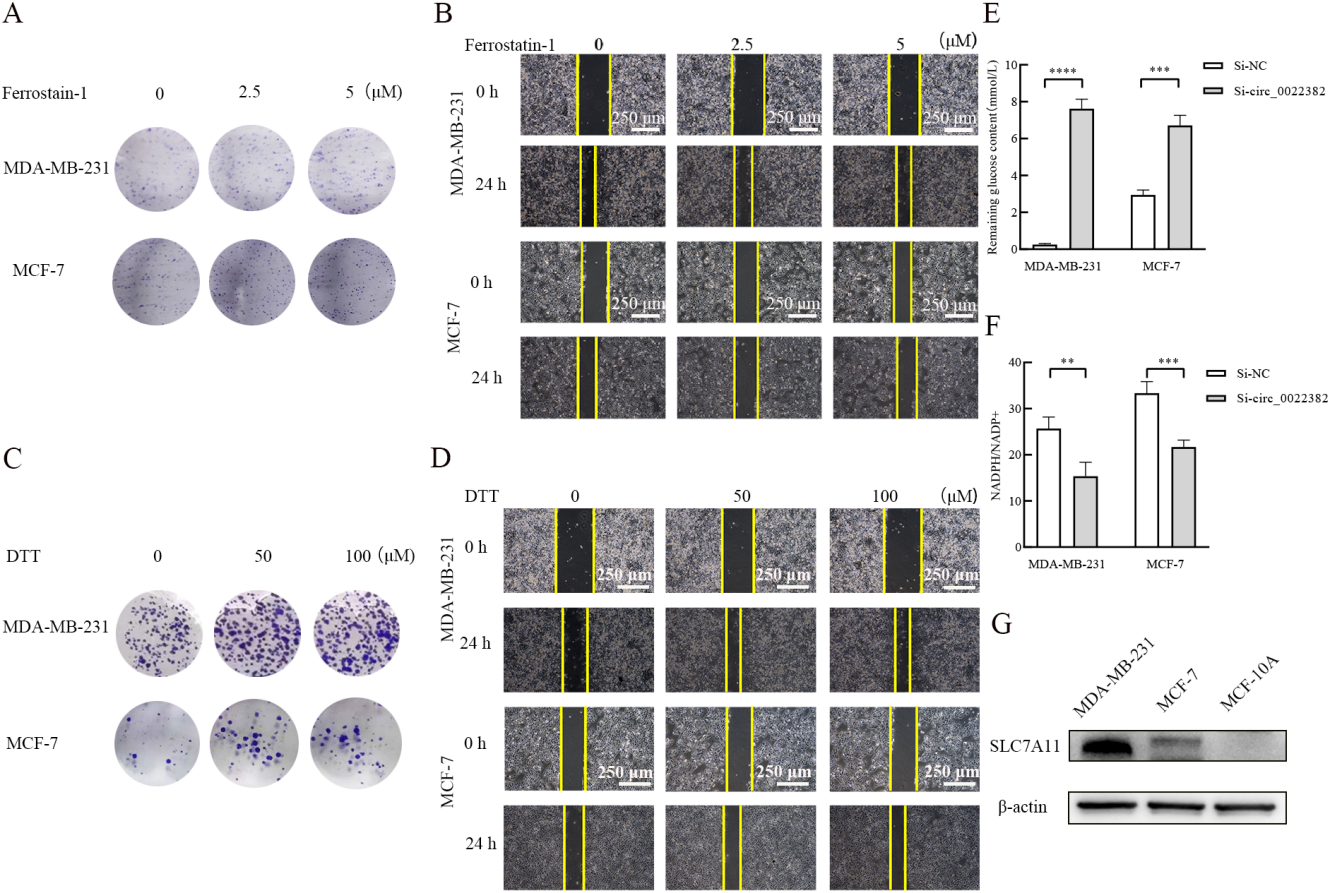
Knockdown of circ_0022382 could contribute to the occurrence of disulfidptosis rather than ferroptosis. **(A, B)** Clone formation and scratch wound healing experiment were used to detect effects of different concentrations of Ferrostatin-1 (ferroptosis inhibitor) on the proliferative and migratory capacity of MDA-MB-231 and MCF-7 cells transfected with si-circ_0022382, scale bar = 250 μm. **(C-D)** Clone formation and scratch wound healing experiment were used to detect effects of different concentrations of dithiothreitol (DTT, cysteine inhibitor) on the proliferative and migratory capacity of MDA-MB-231 and MCF-7 cells transfected with si-circ_0022382, scale bar = 250 μm. **(E)** The concentration of residual glucose in the culture medium supernatant of MDA-MB-231 and MCF-7 cells transfected with si-circ_0022382. ***p<0.001, ****p<0.0001 vs. Si-NC group. Data are the means ± SEM of three independent experiments. **(F)** The ratio of the concentrations of NADPH and NADP^+^ in MDA-MB-231 and MCF-7 cells transfected with si-circ_0022382. **p<0.01, ***p<0.001 vs. Si-NC group. Data are the means ± SEM of three independent experiments. **(G)** SLC7A11 protein expression in MDA-MB-231, MCF-7 and MCF-10A was detected by Western blot.

Then, previous study had reported that SLC7A11 was associated with ferroptosis and disulfidptosis (11, 12), especially disulfidptosis, consistent with PI3K/AKT/mTOR signaling pathway, was related with metabolism of glucose (12, 21), which provided evidence SLC7A11 may indirectly interact with PI3K/AKT/mTOR signaling pathway. To further explore the relationship, we added Ferrostatin-1 (ferroptosis inhibitor) and dithiothreitol (DTT, cysteine inhibitor) respectively in MDA-MB-231 and MCF-7 cells transfected with si-circ_0022382, the results of clone formation and scratch wound healing experiment showed dithiothreitol instead of Ferrostatin-1 could rescue effects of si-circ_0022382 on the proliferation and migration of cells (**Figures 4A–D**), which indicated cysteine accumulated in cells transfected with si-circ_0022382, moreover, the remaining glucose concentration in culture medium of cells transfected with si-circ_0022382 significantly increased (*p*<0.001 or *p*<0.0001; **Figure 4E**) and the ratio of the concentrations of NADPH and NADP^+^ in cells transfected with si-circ_0022382 significantly decreased (*p*<0.01 or *p*<0.001; **Figure 4F**). The above results revealed that knockdown of circ_0022382 in MDA-MB-231 and MCF-7 increased the accumulation of cysteine, meanwhile reduced the consumption of glucose and the production of NADPH within cells, which was consistent with the characteristics of disulfidptosis. In addition, SLC7A11 expression in MDA-MB-231 and MCF-7 was higher than in MCF-10A (**Figure 4G**), indicating that SLC7A11 could also be a potential target.

### 3.5 EIF4A3 promotes the production of circ_0022382 in breast cancer cells

**Figure 5:**
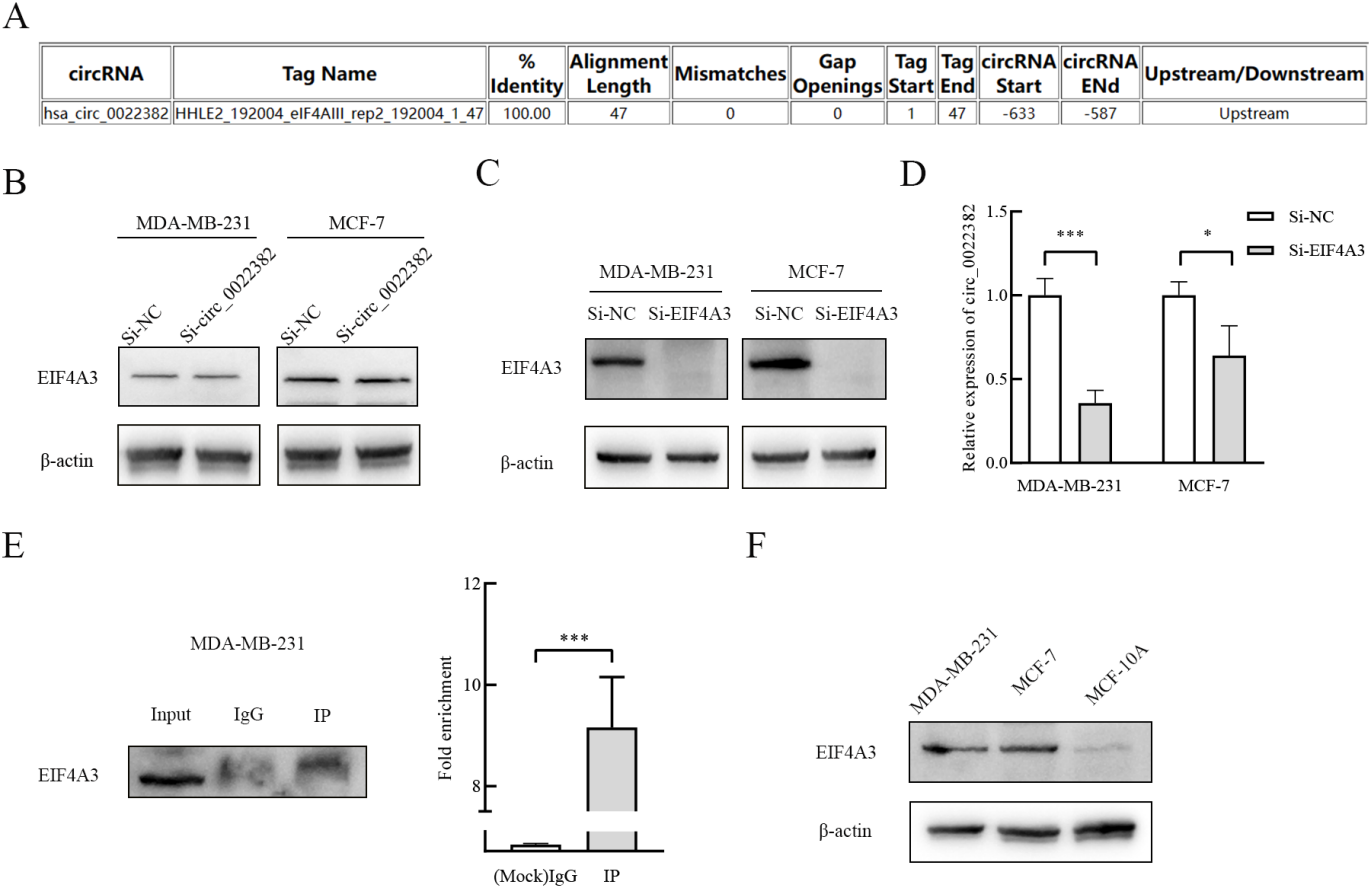
EIF4A3 could promote the production of circ_0022382 in breast cancer cells. **(A)** CircInteractome was used to predict the binding sites of circ_0022382 and EIF4A3. **(B)** EIF4A3 protein expression in MDA-MB-231 and MCF-7 cells transfected with si-NC or si-circ_0022382 was detected by Western blot. **(C)** EIF4A3 protein expression in MDA-MB-231 and MCF-7 cells transfected with si-NC or si-EIF4A3 was detected by Western blot. **(D)** Circ_0022382 expression in MDA-MB-231 and MCF-7 cells transfected with si-NC or si-EIF4A3 was detected by qRT-PCR. *p<0.05, ***p<0.001 vs. Si-NC group. Data are the means ± SEM of three independent experiments. **(E)** The interaction between EIF4A3 and circ_0022382 in MDA-MB-231 was tested by RNA immunoprecipitation (RIP), left was the enrichment of EIF4A3 protein in the whole cell lysate (Input), eluent for magnetic beads incubated with IgG (IgG), eluent for magnetic beads incubated with anti-EIF4A3 (IP), with IgG as a negative control, Input as a positive control, right was the enrichment of circ_0022382 in the IgG group and IP group. ***p<0.001 vs. IgG group. Data are the means ± SEM of three independent experiments. **(F)** The expression of EIF4A3 protein in MDA-MB-231, MCF-7 and MCF-10A was detected by Western blot.

Through CircInteractome database, we found that EIF4A3 had binding sites on the upstream sequences of circ_0022382 (**Figure 5A**), which indicated circ_0022382 could also bind to RNA-binding proteins. To further clarify the interaction between EIF4A3 and circ_0022382, expression of EIF4A3 and circ_0022382 were detected in MDA-MB-231 and MCF-7 cells transfected with si-circ_0022382 and si-EIF4A3 respectively. It is worth noting that EIF4A3 protein expression remained unchanged in cells transfected with si-circ_0022382 (**Figure 5B**), indicating the regulatory effect of circ_0022382 did not depend on EIF4A3 expression, in contrast, circ_0022382 expression dramatically decreased in cells transfected with si-EIF4A3 (*p*<0.05 or *p*<0.001; **Figures 5C, D**), in addition, the results of RNA immunoprecipitation (RIP) showed a prominent enrichment of circ_0022382 in anti-EIF4A3 (IP) group than in IgG group (*p*<0.001; **Figure 5E**), which further demonstrated the role of EIF4A3 in the process of circ_0022382 production in breast cancer cells. We also tested the EIF4A3 expression among breast cancer cells and normal cells, the results showed that MDA-MB-231 and MCF-7 had higher EIF4A3 expression than MCF-10A (**Figure 5F**), which was consistent with high expression of circ_0022382 in breast cancer cells.

### 3.6 Knockdown of circ_0022382 inhibits the growth of breast cancer cells *in vivo*

**Figure 6:**
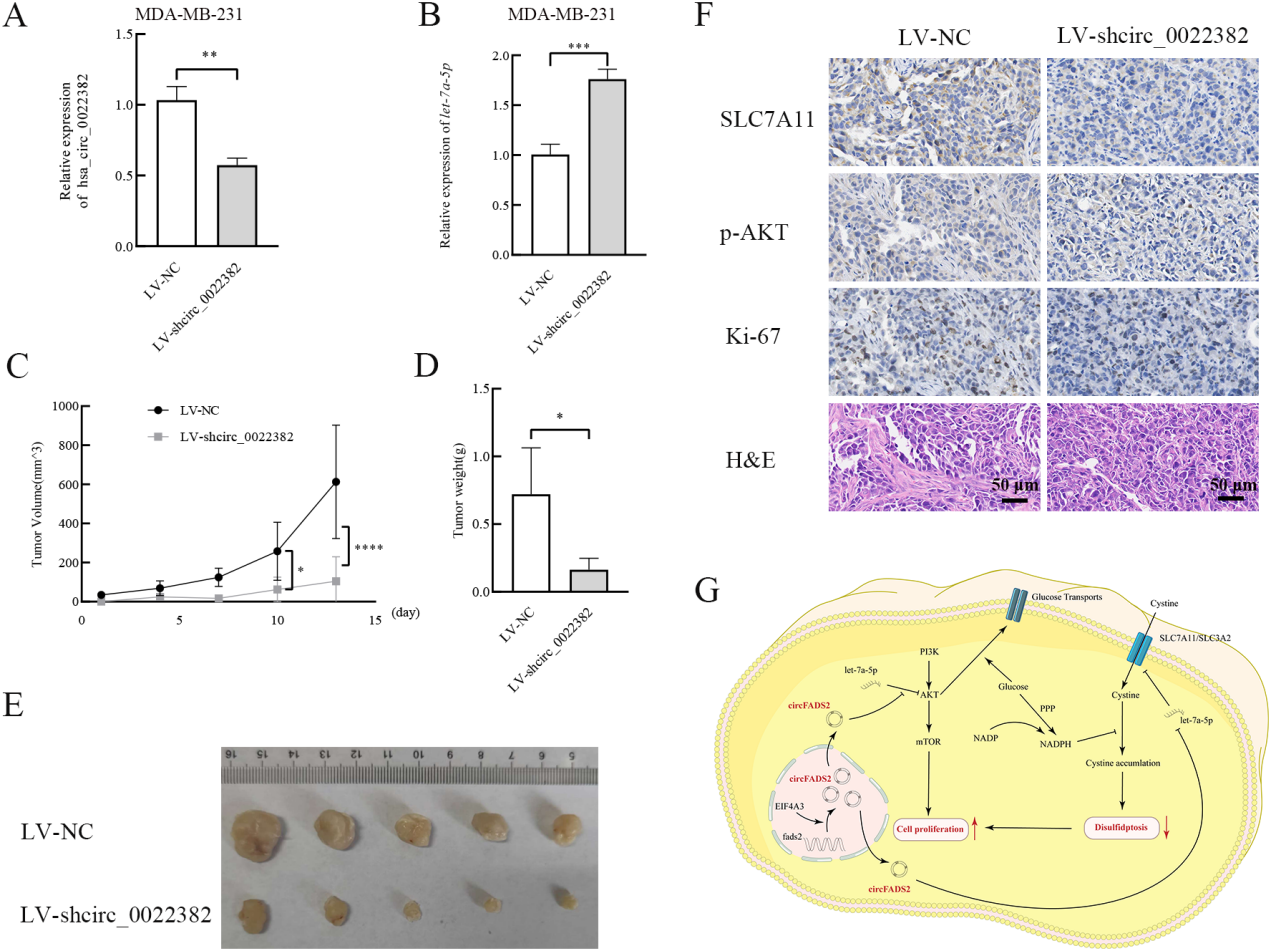
Circ_0022382 promotes the growth and progression of breast cancer *in vivo*. **(A, B)** The expression of circ_0022382 and let-7a-5p in MDA-MB-231 transfected with LV-NC or LV-shcirc_0022382 was detected by qRT-PCR. **p<0.01, ***p<0.001 vs. LV-NC group. Data are the means ± SEM of five independent experiments. **(C)** The volume of tumors derived from LV-NC group mice (n = 5) and LV-shcirc_0022382 group mice (n = 5) was measured every 3 days. *p<0.05, ****p<0.0001 vs. LV-NC group. Data are the means ± SEM of five independent experiments. **(D)** The weight of tumors from the above two groups was measured after two weeks. *p<0.05 vs. LV-NC group. Data are the means ± SEM of five independent experiments. **(E)** The size of tumors from the above two groups. **(F)** Tumor tissues from the above two groups were immunohistochemistry stained with anti-SLC7A11, anti-p-AKT, anti-Ki-67 and H&E; scale bar = 50 μm. **(G)** The mechanism of circ_0022382/let-7a-5p/PI3K/AKT/mTOR signaling pathway and SLC7A11 axis was illustrated by a diagram.

To further confirm the role of circ_0022382 played in the proliferation of breast cancer *in vivo*, we prepared MDA-MB-231 cells transfected with LV-NC or LV-shcirc_0022382, the results of qRT-PCR showed circ_0022382 expression in cells transfected with LV-shcirc_0022382 markedly decreased (*p*<0.01; **Figure 6A**), while let-7a-5p expression significantly increased (*p*<0.001; **Figure 6B**), then the cells were collected and injected into five BALB/C nude mice respectively by subcutaneous tumor-bearing experiment, the results showed the growing rate of tumors in LV-shcirc_0022382 group was significantly slower than in LV-NC group (*p*<0.05 or *p*<0.0001; **Figure 6C**), two weeks later, we observed lighter weights of tumors in LV-shcirc_0022382 group than in LV-NC group mice (*p*<0.05; **Figure 6D**), as shown in **Figure 6E**, effects of circ_0022382 on cell proliferation *in vivo* were consistent with the results of CCK8. Moreover, the tumors were prepared into paraffin slides and the expression of p-AKT, SLC7A11, Ki-67 was detected by immunohistochemistry staining respectively, the results showed p-AKT, SLC7A11 as well as Ki-67 expression decreased in tumors derived from LV- shcirc_0022382 group mice (**Figure 6F**), which were also consistent with the findings *in vitro*. Lastly, the mechanism of circ_0022382/let-7a-5p/PI3K/AKT/mTOR signaling pathway and SLC7A11 axis was depicted in the diagram (**Figure 6G**).

## 4 Discussion

The expression of circ_0022382 in breast cancer tissues and cells were higher than in normal breast tissues and cells, and knockdown of circ_0022382 could inhibit the proliferation and migration of breast cancer cells. Therefore, circ_0022382 may serve as an oncogene of breast cancer. In terms of mechanism, circ_0022382 could bind to let-7a-5p, it was reported that the down-regulated let-7a-5p in breast cancer could regulate the growth and progression of breast cancer cells, for example, the let-7a-5p/DUSP7 axis was associated with paclitaxel resistance (22), the let-7a-5p/GLUT12 axis (23) and the let-7a-5p/STAT3/hnRNP-A1/PKM2 axis (24) were associated with glycolysis in breast cancer. The occurrence of disulfidptosis often happens in cells with high SLC7A11 expression, allowing the accumulation of cystine within cells and then exposes a targetable metabolic vulnerability in cancer (12, 13). However, in this paper, we found expression of SLC7A11 in breast cancer cells transfected with si-circ_0022382 was decreased, which seems not to meet the common condition for disulfidptosis to occur. Of note, in the recent study, Yan et al. drew the conclusion that cells with moderate levels rather than high or low levels of SLC7A11 could undergo disulfidptosis rather than oxidative stress (25), which broke the boundaries of disulfidptosis cognition. As for why dithiothreitol rather than Ferrostatin-1 could rescue effects of si-circ_0022382 on the proliferation and migration of breast cancer cells, this is because both the consumption of glucose and the production of NADPH within cells transfected with si-circ_0022382 markedly decreased, thereby preventing the conversion of cystine to cysteine, which may also cause the accumulation of cystine within cells. Therefore, we have reason to suspect that MDA-MB-231 and MCF-7 have high levels of SLC7A11, and knocking down circ_0022382 will put SLC7A11 at a medium level rather than a low level, but when the circ_0022382 is knocked out, the expression level of the SLC7A11 may be further reduced, reaching a so-called low level, cells are more likely to undergo ferroptosis rather than disulfidptosis at that time, although these speculations need to be further verified. EIF4A3, as an RNA helicase, plays an important role in transcription and post transcriptional regulation (26), and the interaction between EIF4A3 and RNA in cancer has been widely studied (27, 28). In addition, EIF4A3 was found highly expressed in many cancers, such as prostate cancer (29), bladder cancer (30), endometrial cancer (31), so EIF4A3 may be an oncogene in cancer. In conclusion, circ_0022382 may serve as a potential marker for diagnosis and treatment of breast cancer, and its regulatory mechanism needs further exploration.

## Data Availability

Original datasets are available in a publicly accessible repository: The original contributions presented in the study are publicly available. These data can be found here: https://www.ncbi.nlm.nih.gov/geo/query/acc.cgi?acc=GSE165884 and https://www.ncbi.nlm.nih.gov/geo/query/acc.cgi?acc=GSE45498.
